# Contribution of point and small-scaled sources to the PM_10_ emission using positive matrix factorization model

**DOI:** 10.1186/s40201-016-0265-8

**Published:** 2017-01-14

**Authors:** Zohre Farahmandkia, Faramarz Moattar, Farid Zayeri, Mohamad Sadegh Sekhavatjou, Nabiollah mansouri

**Affiliations:** 1 Department of Environmental Engineering, Faculty of Environment and Energy, Science and Research Branch, Islamic Azad University, Tehran, Iran; 2 Department of Biostatistics, Faculty of Paramedical Sciences, Shahidbeheshti University of Medical Sciences, Tehran, Iran; 3 Department of Environmental Engineering, Islamic Azad University, Ahvaz Branch, Ahvaz, Khouzestan Iran

**Keywords:** Characterization, PM_10_, Heavy metals, Positive Matrix Analysis

## Abstract

**Background:**

The positive matrix factorization is a powerful environmental analysis technique which has been successfully utilized to assess air-born particulate matter source contribution. The new version of this model (PMF5) has two additional estimation error methods and some other useful advantages compared to the previous versions. In the present study, the capability of PMF5 for identification and contribution of small size particle source to the ambient particulate matter was evaluated.

**Methods:**

The study area is surrounded by three industrial complexes and 2 locations of dumped tailing soils of mining activities and related manufactures. Ambient particulate matter were sampled at 2 sites in the urban area of Zanjan (Iran) and 196 collected samples were analyzed for 15 chemical elements.

**Results:**

At downtown, the identified factors (and their contributions to particulate matter) were: soil particles (40.36%), fuel combustion and traffic (26.8%), tailing soils (lead and zinc) (21.32%), and nickel and industrial emission(5.7%). The identified factors at residential site of studied area (and their contributions to particulate matter) were general industrial emission (28.2%), tailing soils (lead and zinc) (39.2%), soil (25.8%), cadmium and general pollutants (6.7%).

**Conclusion:**

The results of modeled data by PMF 5 indicated that the applied model could identify the dumps of tailing soils as a separated factor. The other particulate matter sources in the studied area were traffic, fuel combustion, soil particles and industrial pollutants.

## Background

The correlation between high concentrations of airborne particulate matter (PM) and morbidity and/or mortality has been shown in many studies [1]. Air-born particles with aerodynamic diameter less than 10 μm (PM_10_) can penetrate into the lungs and enter toxic chemicals into human body [2]. Construction of industrial centers near the cities, traffic, transportation, mining, agricultural and construction activities are the main anthropogenic sources of air-born particles in the air. The size and composition of the particles determine the degree of penetration into the lungs and harmful effects on the human health [3]. These parameters depend on the sources of the particles, therefore, source identification and apportionment of the air-born particles are tow basic measures in the urban air quality management systems. Particulate matters contain organic and inorganic chemicals. Among inorganic compounds, heavy metals are the most important ones owing to their harmful effects on the environment and human health.

For that reason, many source apportionment methods have been applied based on the statistical evaluation of data, emission inventories or dispersion models, and evaluation of monitoring data. Chemical mass balance (CMB), factor analysis, principle component analysis (PCA), multiple linear regression methods and positive matrix factorization (PMF) have been used by researchers in many studies [4]. PMF is a widely used multivariate method which can find the main sources of particles without prior knowledge of the sources. The model fundamentally resolves the identities and contributions of components in an unknown matrix. The most important advantage of this model is that it has potential to incorporate variable uncertainties associated with environmental sample measurements [5].

There are many studies on source apportionment and identification of atmospheric particulate matter using PMF. Most of these studies were conducted with PMF versions of 1,2 and 3 at numerous locations around the world, such as Barcelona(Spain), Belgrade(Serbia), Brisbane(Australia), scuttle(USA) phoenix, AZ (USA), Atlanta, GA(USA), Hanoi(Vietnam) Guenon(Italy) Augsburg(Germany) Rochester(USA), Karachi(Pakistan) Atlanta(USA) Thessaloniki(Greece), Queensland(Australia), Erfurt(Germany), Gosan (Island), [2, 4, 6–19]. According to the previous studies carried out in many urban areas, the main sources of particles were crustal material, road dust, industrial activities and traffic.

Mining activities such as concentrated soil preparation for material (metal) extraction and metal melting are accompanied by the production of great amounts of tailing soils. These soils contain fine particles which can readily re-suspend into the atmosphere by wind.

These particles contain a high percentage of metals especially heavy metals such as cadmium,lead, and chromium whose distribution on the ground can pose serious threats to human public health.

Iran produces three percent of the total world reserves of lead and zinc and is the fourth producer of lead and zinc concentrated soil in Asia following china, Kazakhstan and India. Asia produces about %45 of the world lead., Angooran which is the largest zinc and lead mine in Iran is located in Zanjan province [20]. Due to its proximity to Zanjan, more than 100 factories of concentrated soil and ingots of lead and zinc have been established around Zanjan. These factories use the raw material from Angooran mine, and even in recent years, industrialists are importing raw materials from other provinces. This development of zinc and lead industries in a small area results in the production of million tons of tailing soils which are accumulated around the factories on the open grounds without any environmental considerations. These soils are produced from filtering of acid leached concentrated soils with very fine particles which are named filter cakes. These particles can emit and re-suspend into the atmosphere easily by wind and move towards the city center and residential zones and threat the public health. Emission of these particles and their settlement on the agricultural grounds can cause soil and groundwater pollutions and enter into food chain.

In addition to zinc and lead industries, there are several small and large scale industrial areas. The city is surrounded by three industrial complexes (Industrial Complex No. 1 in the North-west, Industrial Complex No. 2 in the southwest, and Zinc Industrial Complex in the south- west). The largest lead production factory in Iran (National Iranian Lead and Zinc Company) is also located in the East of Zanjan. In these industrial complexes, more than 200 manufacturers are active and release large amounts of air pollutants into the atmosphere. In recent years, the contribution quantity of open dumped tailing soils to air born PM in Zanjan is considered as the main concern for environmental and governmental organizations.

Most of the published studies have focused on large-scaled and non-point particle sources such as combustion, traffic, soil and industrial activities. However, limited research has been done on the contribution of small-scaled and point sources in particle emission. The aim of present study was an attempt to report the source identification and apportionment of emitted particles with an emphasis on the unburied tailing soils as a small-scaled and point source near an urban area using EPA-PMF 5. Since chemical characterization of these soils is very similar to that of crustal soil, the capability of EPA-PMF 5 to differentiate between these two similar particle sources is another objective of this study. Although the fifth version of EPA-PMF model was first introduced in 2014, there are few studies which have used this new version [21].

Compared to EPA-PMF 3, EPA-PMF 5 has two additional error estimation methods that are very useful in the determination of the number of factors [21]. In most of the studies in this field, there is a relatively small number of discussions about the process of factor number determination. In the present study, attempts have been made to determine the number of factors considering error estimation methods (displacement and bootstrapping) based upon the methods presented by Brown et al. [22].

## Methods

### Description of the study area

**Fig. 1:**
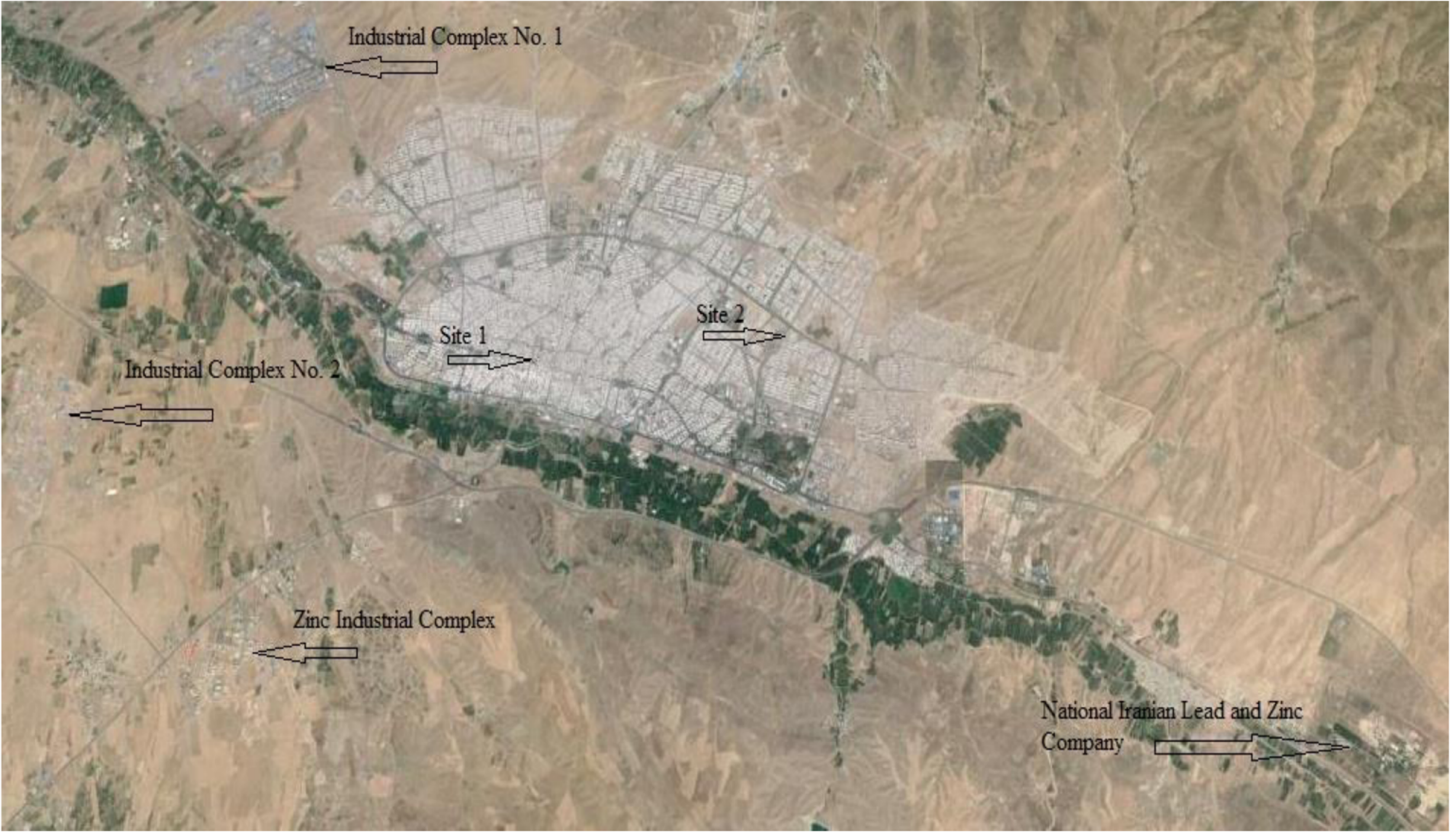
A map of zanjan with industrial complexes and open dumps of tailing soils and sampling sites

Zanjan (the capital of Zanjan province) is situated in the north-west of Iran (latitude 36 41 N longitude 48 27 E) at an average height above the sea level of 1620 m. This city had a population of about 400,000 in an area of 81 km^2^ in 2015. The climate of Zanjan is cold semi-arid, with hot dry summers and cold moist winters. Mean annual air temperature is 10 °C, mean annual rainfall is 295 mm and the prevailing wind is eastern with an average speed of 3 m/s. The surrounding areas of Zanjan are characterized by light to heavy industrial complexes. There are two dumps of tailing soil nearby. The first dump is located beside the Zinc Industrial complex with an area of around 1 Km^2^ and contains more than three million tons of tailing soil in which about 100 zinc factories discharge their tailing soils. The second is situated beside the National Iranian Lead and Zinc Company with an area of less than 1 Km^2^. The location of industrial complexes and open dumps of tailing soils are shown in Fig. 1.

### Data collection

A high-volume sampler (TCR- Tecora) was used to collect ambient PM_10_ samples at the flow rate of 16.7 L/min. Daily 24-h PM_10_ samples were collected weekly from July 2013 to July 2015 (totally192 samples). The sampling operation was conducted according to the USEPA-IO2.1 method (1999) [23]. Fiberquartz filters were used for sampling PM_10_. Then, the samples were immediately transformed to the laboratory for digestion operation. A microwave digester (sineo-model mds-10) was employed for digestion operation using microwave method of USEPA-IO-3.1 (1999) [24]. The digested samples were analyzed by inductivity coupled plasma method of USEPA-IO-3.4(1999) [25]. ICP-OES (Spectro) was utilized to analyze the traces of Ca, Ni, Pb, Zn, Al, As, Sb, V, Hg, Cd, Cr, Cu, Ti, Mn and, Fe in PM_10_ samples. The operation parameters of ICP-OES are demonstrated in Table 1. The weight of PM_10_ in the samples was measured using the gravimetric method.

**Table 1:** The operation parameters of ICP-OES

Parameter	Values
RF generator (W)	1400
Plasma torch	auxiliary
nebulizer gas	Argon
Plasma gas flow rate (l/min)	14.5
Auxiliary gas flow rate (l/min)	0.9
Nebulizer gas flow rate (l/min)	0.85
Sample uptake time (S)	240 total
Rinse time of (S)	60
Initial stabilization time (S)	Preflush:60
Measurement replicate	3
Element (λ/nm)	As below
Frequency of RF generator (MHz)	resonance frequency: 27.12 MHz
Type of detector Solid state	CCD
Type of spray chamber Cyclonic	Modified Lichte

In order to calculation of sample size in this research, 30 samples in each site were collected and after calculation of standard deviation of metals concentrations, the sample size was calculated using statistical formula.

Because low standard deviation of data, the achieved sample size was small. On the other hand, in order to achieve a valid solution with PMF model, the sample size should be at least 100 (19).

In this research 100 sample were collected in each study site. Four samples were removed because the local storm and modeling was carried out with 96 samples of each site and totally 192 samples.

Two monitoring sites were selected in the urban area. Site one is downtown where the traffic load is heavy. This zone is the center of commercial activities. It is near the south of Zanjan-Tabriz highway. The air pollutants which are originated from industrial complexes affect the quality of air in this site. Site two is the north-east of Zanjan. This zone is merely residential and the traffic load and commercial activities in this zone are low. The distance between these two sites is about four kilometers.

### Data analysis method

Positive matrix factorization (EPA-PMF 5), a multivariate receptor based model, was used for source apportionment and characterization of the collected PM_10_ [26]. A PMF model assumes that there are p factors (sources) which can be involved in a receptor site and can be stated with the following equation:

1 $$ {\mathrm{X}}_{\mathrm{ij}}={\displaystyle {\sum}_{k=1}^p\left({g}_{ik}\times {f}_{kj}\right)+{E}_{ij}} $$

Where,

*X*_*ij*_ Concentration of species J in ith sample

*g*_*ik*_ Contribution of kth factor to the ith sample

*f*_*kj*_ Fraction of kth factor that is species j or chemical composition profile of factor K

*E*_*ij*_ Residual for the jth species on the ith sample

The contributions of factor (*g*_*ik*_) and source profiles (*f*_*kj*_) are estimated by the PMF model by minimizing the objective function:

2 $$ Q={\displaystyle {\sum}_{i=1}^n}{\displaystyle {\sum}_{j=1}^m}\left(\frac{x_{ij}\_{\displaystyle {\sum}_{r=1}^p}{g}_{ik}\times {f}_{kj}}{u_{ij}}\right) $$

*u*_*ij*_: Uncertainty of the jth species of the ith sample

Q: objective function.

The main aim of EPA PMF is to minimize the sum of squares of standardized residuals or Q. In EPA PMF_5,_ two versions of Q are applied and displayed for the model runs.

1. Q_true_ is the goodness-of-fit parameter calculated including all points.
2. Q_robust_ is the goodness-of- fit parameter calculated excluding points not fit by the model which are the samples with uncertainty-scaled residual greater than 4. The difference between these two Qs is the degree of the impact of the data points with high-scaled residuals [21].

Two input files of the data and uncertainty values were prepared according to the described method in the PMF5 manual. PM_10_ concentrations were included in the data file (first input file) as the independent variable [11]. The uncertainty values were calculated as below and were included in the uncertainty file (second input file).

Determination of the uncertainty for each of the measured data is the pre-requisite for the application of PMF. In PMF, the weight of missing and below-detection-limit data would decrease with appropriate uncertainty [27]. In order to determine the uncertainties in the data, the standard deviation of repeated analysis of standard reference materials was used and the detection limit (MDL) of each species was calculated. PMF has the ability to underweight the missing data and values below the detection limit, and can reduce the influence of extreme values using robust mode [1]. The uncertainties of the species were determined according to the recommended methods and equations in the EPA-PMF5 manual [21].

The data with concentrations below MDL, substituted by 1/2 MDL and 5/6 MDL was used as the corresponding uncertainty value [5].

If the concentration was greater than MDL, the following equation was used [21].

$$ \mathrm{U}\mathrm{N}\mathrm{C}=\sqrt{{\left( Error\  fraction\times concentration\right)}^2+{\left(MDL/2\right)}^2} $$

As described by Norris et al. [21] and detailed in Paatero et al. [28], EPA PMF has 2 main error estimation methods: displacement (DISP), Bootstrapping (BS), as well as a useful tool for rotation that is named F peak [21, 28].

DISP includes the effects of rotational ambiguity and does not affect random errors in the data. BS includes the effects of random errors and partially-rotational ambiguity.

In this study, the number of factors was determined on the basis of variations in values of Q _true_ and Q _robust_ and IQ _expected_. The identified sources of the trace metals in PM_10_ were interpreted physically based on the field information and wind directions.

The S/N calculation in PMF 5 has been revised which is described in the EPA PMF 5 user guide in details. In order to reduce the weights of the species with low S/N in the solution, the species with S/N ratio less than 1 were categorized as weak variables [21]. Most of the species have S/N higher than 8, the reason for this high S/N is that the species were analyzed in PM_10_, hence, most of the concentrations were high.

## Results and discussion

A total of 192 samples were collected from site 1 (high traffic load) and site 2 (residential) (96 samples in each station). The median of PM_10_ in site 1 was 61.8 (μg/m^3^) and in site 2 was 31.3(μg/m^3^). The reason of this result is the traffic effect in site 1.

Table 2 shows the correlation between the daily spaces concentrations of two sites. The correlation coefficient values(r) shows that there is very weak correlation between spaces concentrations in two sites. It means that the patterns off particle emission and the origins of metals in two sites are different. It also shows that the locations of studied sites are suitable.

**Table 2:** The correlation coefficients of elements between 2 sites

Element	Correlation coefficient	Element	Correlation coefficient
PM_10_	0.250	Hg	0.146
Al	0.330	Mn	0.082
As	0.038	Ni	0.093
Ca	0.451	Pb	0.110
Cd	0.370	Sb	0.083
Cr	0.083	Ti	0.002
Cu	0.149	V	0.166
Fe	0.350	Zn	0.012

Without mathematical tools or models, interpretation of the species data, because of high variation in metals and their different concentrations in different days and seasons is impossible.

**Fig. 2:**
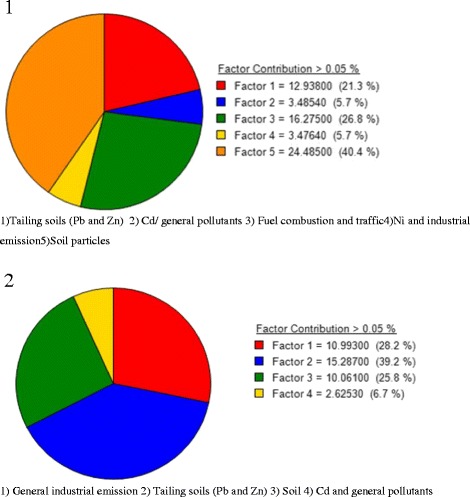
Five-factor solution contributions 1) site 1, 2) site 2

**Fig. 3:**
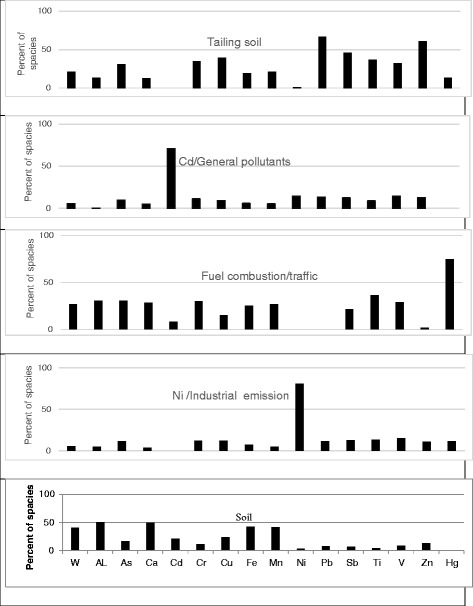
Factor profiles for a five-factor solution at site 1(W: ﻿PM_10_)

**Fig. 4:**
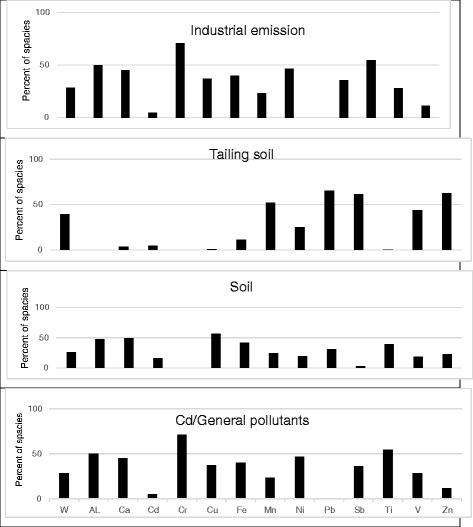
Factor profiles for a four-factors solution at site 2(W: PM_10_)

Tables 3 and 4 list the species quantified along with their max., min., median and percentiles of the species concentrations. The species with concentrations lower than MDL in most of the samples and/or S/N ratios lower than two were categorized as Bad. PM_10_ and As were categorized as weak in the modeling process of site1. In order to model the collected data of site 2, the PM_10_ mass and V were categorized as weak, and As and Hg were categorized as Bad. Modeling operations of the data by PMF were started with 100 runs and the run with Q_Min_ was retained for four-five-and-six factor solutions and the other estimation methods. The BS was run with 100 bootstraps, 3 block size and minimum correlation R-value of 0.6. The results demonstrated that most of the species were well modeled. Figure 2 shows the pie charts of factor contribution to PM_10_ mass in four-five-and-six factor solutions. The PMF factor profiles and related PM10 contributions in the studied sites are presented in Figs. 3 and 4.

**Table 3:** Particle (PM_10_) and elemental concentrations in the samples of site 1(μg/m^3^)

Species	Max.	Min.	25th	Median	75th
PM_10_	100.4000	16.7000	50.1250	61.8500	77.2000
Al	0.4852	0.10523	0.17078	0.25494	0.34788
As	0.0002	0.00008	0.00018	0.00019	0.00020
Ca	2.1874	0.45917	0.73649	1.04812	1.54829
Cd	0.0090	0.00017	0.00117	0.00239	0.00348
Cr	0.0238	0.00972	0.01239	0.01310	0.01500
Cu	0.0165	0.00708	0.00867	0.00961	0.01238
Fe	1.2860	0.33425	0.53150	0.72310	0.95696
Mn	0.0516	0.01250	0.02232	0.03038	0.03874
Ni	0.0202	0.00102	0.00173	0.00392	0.00601
Pb	0.1022	0.01587	0.02848	0.04720	0.06644
Sb	0.0436	0.01893	0.02202	0.02523	0.02983
Ti	0.0050	0.00161	0.00207	0.00257	0.00293
V	0.0010	0.00004	0.00010	0.00010	0.00011
Zn	0.3581	0.07383	0.14846	0.20114	0.25309
Hg	0.0091	0.00145	0.00236	0.00343	0.00517

**Table 4:** Particle (PM_10_) and elemental concentrations in the samples of site 2(μg/m^3^)

Species	Max.	Min.	25th	Median	75th
PM_10_	67.9000	25.0000	31.3000	36.0000	45.8000
Al	0.4080	0.0921	0.1118	0.1844	0.3056
As	0.0005	0.0002	0.0003	0.0003	0.0003
Ca	2.3234	0.4055	0.6769	0.9463	1.3843
Cd	0.0098	0.0010	0.0017	0.0057	0.0085
Cr	0.0011	0.0000	0.0003	0.0005	0.0006
Cu	0.0384	0.0077	0.0097	0.0125	0.0218
Fe	2.0891	0.3875	0.4926	0.6517	0.9483
Mn	0.0418	0.0126	0.0182	0.0234	0.0291
Ni	0.0057	0.0011	0.0018	0.0024	0.0033
Pb	0.0809	0.0150	0.0213	0.0328	0.0481
Sb	0.0076	0.0002	0.0016	0.0022	0.0024
Ti	0.0050	0.0005	0.0016	0.0025	0.0040
V	0.0002	0.0001	0.0001	0.0001	0.0001
Zn	0.3409	0.0784	0.1377	0.1619	0.2101
Hg	0.0095	0.0002	0.0012	0.0015	0.0020

### Identified sources of studied sites

#### Site 1

Four-five-and-six factor solutions were tested with the data of site one and the results are shown in Figs. 2 and 3. In the four-factor solution, the factors (and their contributions) included:

- Soil particles (40.36%)
- Fuel combustion and traffic (26.8%)
- Tailing soils of Pb and Zn (21.32%)
- Ni and industrial emission (5.7%)

At five-factor solution, an additional factor with key space of Cd was separated from the soil and tailing soil factors. At all solutions with four-five-and-six factors, all key species were well predicated. It means that their r^2^ of the observed/predicated scatter plots were greater than 0.8 [22].

The achieved values of Q/Q _expected_ were 1.15, 0.63 and 0.49 at four-five-and-six factor solutions respectively. The decrease of Q/Q _expected_ in moving from four to five factors was higher than the value in moving from five to six factors. It indicates that there may be too many factors being fit, therefore, five factors may be the optimal solution [22].

In BS runs with four-factor solution, factors were mapped in 96%, 99%, 95%, and 100% of the runs and in DISP run there were no swaps in all of the dQ_max_ levels and the largest observed drop of Q during DISP was 0.1%. At five factors solution, factor four was mapped in 96% of the runs and, the others were mapped in 100% of the runs and in DISP run there were no swaps in the first two dQ_max_ levels (4 and 8) and the largest observed drop of Q during DISP was 0.09%.

In six-factor solution, the factors had larger swaps in DISP run and BS factors were not mapped with base factors. These results showed that there were a significant rotation ambiguity and a random error in this solution.

In this present study, the error estimation interval ratios of the key species of each factor as presented by Paatero et al. [28] were used to compare the results. The DISP and BS intervals ratios at four-and five-factor solutions were almost equal to each other and quite lower than six-factor solution. These values indicate that there are little rotational ambiguity and low estimation error in this solutions. Higher interval ratios indicate higher uncertainty for key species [22].

With regards to the results of BS mapping, DISP swaps and interval ratios of three solutions, it can be concluded that five-factor solution is the best and more stable than the other solutions.

#### Site 2

Site 2 is located in a residential zone in the north-east of Zanjan. The results of the modeled data of site 2 are demonstrated in Figs. 2 and 4.

The PMF model was run with four-five-and-six factor solutions and the BS and DISP were also run for the estimation of the errors in each solution with the parameters similar to those of the site1 data modeling.

At four-factor solutions, the identified factors (and their contributions to PM_10_) are as follows:

- General industrial emission (28.2%)
- Tailing soils of Pb and Zn (39.2%)
- Soil (25.8%)
- Cd and general pollutants (6.7%)

At five-factor solution, a new factor appeared with high contribution of Cd. The values of the regression coefficient in the observed/predicted scatter plots for key species of the identified factors were higher than 0.8. The achieved values of the Q/Q _expected_ at different factor number solutions were 1.38, 0.98 and it was found to be 0.7 at four-five-and-six factor solutions respectively. The decrease in Q/Q _expected_ when moving from four-factor solution to five-factor solution was significantly more than when moving from five to six factors.

In order to interpret the number of the factors more accurately, it is necessary to analyze the solutions using DISP and BS methods. At four-and-five factor solutions, there were not any swaps in DISP runs in dQ_max_ = 4. The values of dQ% in both solutions were lower than 1%. In four-factor solution, all factors were mapped in more than 80% of the runs and in five-factor solution, factor 3 was mapped in 41% of the runs. These results showed that there is a significant random error at five-factor solution in this sampling site. The results of the BS and DISP at 6 factors solution showed both random error and rotational ambiguity in this solution. The calculated BS and DISP interval ratios of the key species in 95% of the cases at four-factor solution were lower than that at five-and six-factor solutions.

These results indicate that the uncertainty of the key species could increase the factors. These results also indicated that four-factor solution is the most interpretable solution as discussed above due to the lowest random errors and rotational ambiguity.

### Rotation solution

In modeling with EPA-PMF, a useful method of rotating solution is F peaks tool. In this method, the rows and columns of F and Q matrices will be added and/or subtracted from each other at different F peaks, and then the %dQ will be calculated by PMF program [5].

In the present study, the F peak was run for the best solutions in both sites in order to introduce the rotations to the solutions. The F peak strengths values were adjusted between 1 and-1 and the %dQ was examined as a function of F peak. The results showed that the non-rotated solutions (F peak =0) were judged most interpretable with corresponding Q values.

### Identification of the sources

In this study, four common sources were identified in both sites. The identified sources were almost similar in both sites, but their contributions to the particulate matter (PM_10_) were different. The order of factors in site 1 is different from the order in site 2. In site 1 re-suspended surface soil is in the first order but in site 2, tailing soils of Pb and Zn is the first. These differences are reasonable considering the low values of correlation coefficients.

The characteristics of the identified sources are briefly presented below.

### Tailing soil (Pb, Zn) source

As mentioned in the introduction, Zanjan is the center of zinc and lead production in Iran. The activity of the related industries is associated with tailing soil production which is dumped unpaved and without any environmental considerations. The two main locations of tailing soils are nearby Zanjan. One of them, with about 3000000 million tons of tailing soils, is located in the southeast with a distance of 5 km from the city beside the complex of zinc factories (zinc industrial complexes). The second is located beside the National Iranian Lead and Zinc Company in the east of zanjan, Zanjan-Tehran road located 12 kms away from Zanjan. These locations are shown in Fig. 1. Re-suspension of these tailing soils by wind must have contributed to this factor.

**Fig. 5:**
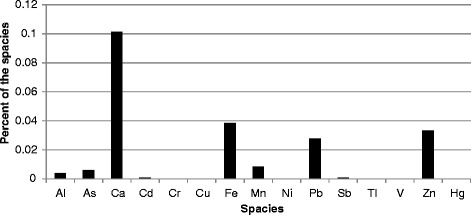
The percent of the trace metals in the collected tailing soil samples

The tailing soil factor is dominated by Pb, Zn, and Fe. In order to determin e the concentration of the trace metals in tailing soils, 20 samples were collected and analyzed using the same analytical method for PM_10_ samples. The presented results in Fig. 5 show that Pb, Zn, Ca and Fe are enriched in the dumped tailing soils. The percentages and concentrations of these species in the identified factor of tailing soils were significantly high too. The contributions of the tailing soils factor in the PM_10_ of sites 1 and 2 were 21.32% and 39.2% respectively. The main reason is the location of tailing soil depot of National Iranian Lead and Zinc Company at east and the direction of prevailing winds.

### Fuel combustion/traffic

The other identified source was fuel combustion/traffic. This source is characterized by high concentrations of Fe, Ni, V, Cu, Zn and Ca [8, 11, 12]. In the present study, the fuel combustion factor contained significant loadings of the above-mentioned metals. The emission from gasoline vehicles and diesel and oil combustion in stationary and moved sources must have contributed to this source. In the studied sites, the separated sources of traffic and industrial fuel combustion sources were not identified. In Iran, lead-free gasoline is used, therefore, the concentration of lead in this factor was low. It has been found that Pb, Zn, and Cu are indicators of traffic emission [11]. Cu, Fe, and Zn are emitted from brake-wear, and vehicle tires are the main source of Zn in road traffics [8].

The contribution of fuel combustion/traffic to the PM_10_ at site 1 was about %26.8. This contribution was ordinary due to the high traffic load in this site. In the residential area (site 2), general pollutants had a contribution of about 6.7%.

### Soil or crustal source

One of the identified sources was represented by Al, Ca, Fe, Zn, and Mn which could typically be soil sources. Re-suspension of the particles from barren soils and arid lands is the major origin of these elements in the PM_10_. The dearth of water in recent years has intensified the resuspension and dispersion the particles in the city. Unpaved roads and construction sites contribute to this factor and also produce particles carrying these crustal elements.

### Industrial emission

The factor, identified as industrial source, includes several elements such as Fe, Mn, Al, Cr, Ni, Hg, and Zn. As mentioned before, Zanjan is surrounded by three main industrial complexes and zinc and lead factories which are regarded as the major industrial centers around the city.

Non-ferrous metallurgy industries such as copper-smelting, lead recycling from used batteries, and electrical industries are the other sources of air pollution in the studied areas.

The contribution of the industrial factor to PM_10_ in the samples collected from sites 1 and 2 were 5.7% and 28.2% respectively. The contribution of Ni to the factor of industrial emission was higher than that of the other metals at site 1. There are tens of plating units around the city center of Zanjan where Ni, Cr, and other plating metals are used and can be the source of Ni in the air.

### The wind directions

**Fig. 6:**
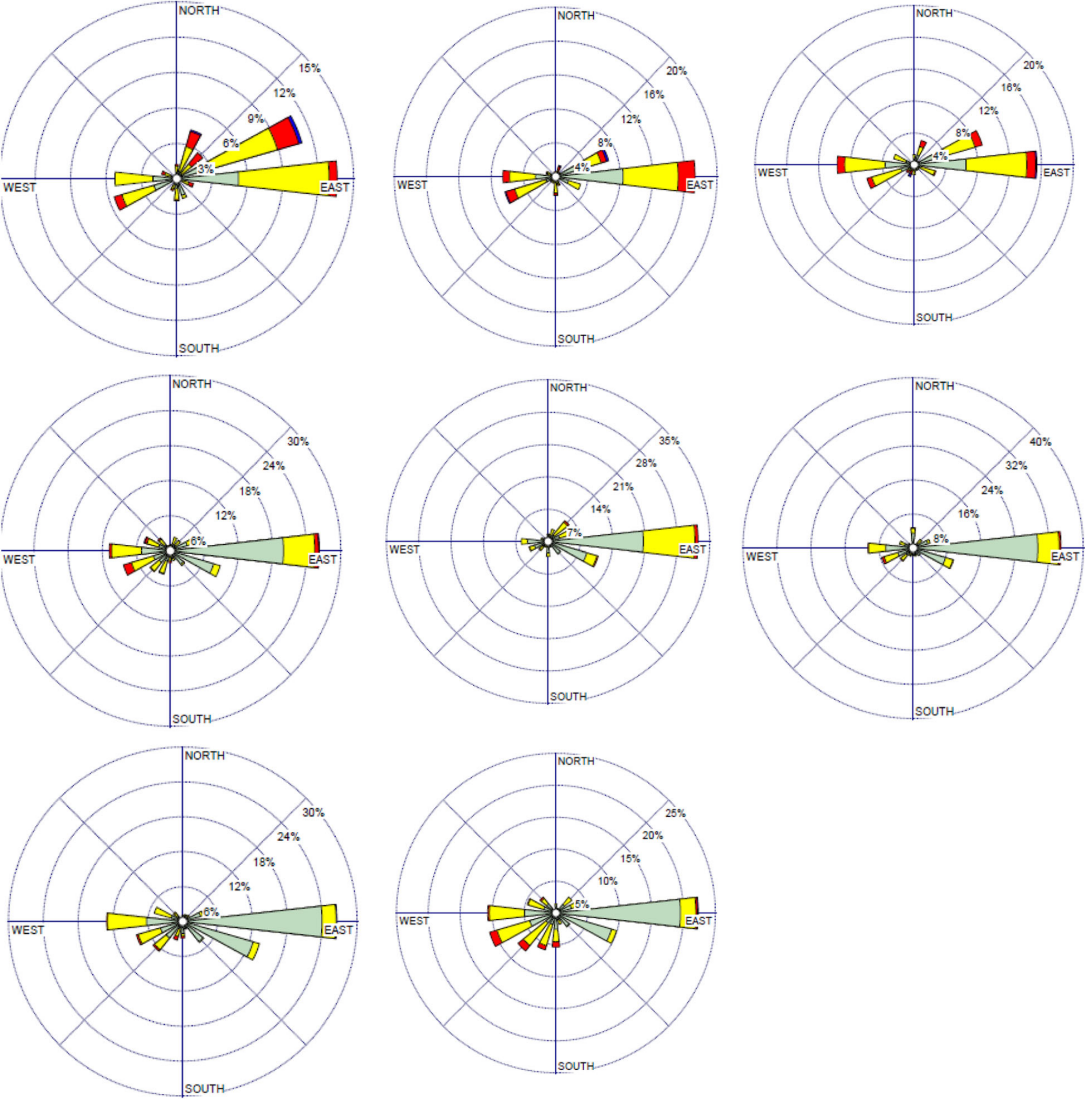
The wind rose of Zanjan in the 8 sampling seasons

The wind rose of Zanjan in the seasons of sampling period is presented in Fig. 6. This figure shows that the prevailing winds were at first, E to W and then SE to NW. The contribution of tailing soil to particle emission in site 2 is higher than in site 2. It means that the particulate matter which is re-suspended from tailing soil dumps beside the National Iranian Lead and Zinc Company at east moves towards the city by wind. In addition, general industrial pollutants are spread in the atmosphere of the city.

## Conclusion

The results of the present study showed that the identified tailing soil factor is one of the major sources of PM_10_ in Zanjan with a contribution of 21.32% downtown and 39.2% in the residential areas. The two described dumps of the tailing soil whose chemical structures were relatively similar to those of soil particle source were limited to two points in a small area. The EPA PMF5 could successfully identify and apportion this source using a small sample size of 96 samples in each site. The identified factors and their quantities proved to be logical given wind direction.

## Abbreviations

BS: Boot strap
CMB: Chemical mass balance
DISP: Displacement
EPA PMF5: Environmental protection agency- positive matrix factorization-virgin 5
ICP-OES: Inductively coupled plasma- optical emission spectrometry
MDL: Method detection limit
PCA: Principle component analysis
PM_10_: Particles less than or equal to 10 micrometers in diameter
UNC: Uncertainty
USEPA: United States Environmental Protection Agency
